# Advances in Atomic Force Microscopy: Imaging of Two- and Three-Dimensional Interfacial Water

**DOI:** 10.3389/fchem.2021.745446

**Published:** 2021-09-22

**Authors:** Duanyun Cao, Yizhi Song, BinZe Tang, Limei Xu

**Affiliations:** ^1^ Beijing Key Laboratory of Environmental Science and Engineering, School of Materials Science and Engineering, Beijing Institute of Technology, Beijing, China; ^2^ International Center for Quantum Materials, School of Physics, Peking University, Beijing, China; ^3^ Collaborative Innovation Center of Quantum Matter, Beijing, China; ^4^ Interdisciplinary Institute of Light-Element Quantum Materials and Research Center for Light-Element Advanced Materials, Peking University, Beijing, China

**Keywords:** interfacial water, atomic force microscopy, structure and dynamics, liquid/solid interface, machine learning

## Abstract

Interfacial water is closely related to many core scientific and technological issues, covering a broad range of fields, such as material science, geochemistry, electrochemistry and biology. The understanding of the structure and dynamics of interfacial water is the basis of dealing with a series of issues in science and technology. In recent years, atomic force microscopy (AFM) with ultrahigh resolution has become a very powerful option for the understanding of the complex structural and dynamic properties of interfacial water on solid surfaces. In this perspective, we provide an overview of the application of AFM in the study of two dimensional (2D) or three dimensional (3D) interfacial water, and present the prospect and challenges of the AFM-related techniques in experiments and simulations, in order to gain a better understanding of the physicochemical properties of interfacial water.

## Introduction

Interfacial water is ubiquitous in nature and closely related to various issues both in fundamental research and technological applications, such as the dissolution of salts (Wandelt and Thurgate, 2002), desalination of seawater (Somorjai and Li, 2010), biological reactions (Tanaka et al., 2002), nanoconfined amorphous water (Manni et al., 2019) and lubricant systems (Urbakh et al., 2004). To gain a deep understanding of these important processes, the investigation of the structure and dynamic process like the rearrangement and growth mechanism of interfacial water on heterogeneous surfaces should be conducted. The symmetry of interfacial water is complex, for it shares boundaries with other substances. Therefore, it is more difficult to investigate interfacial water compared with bulk water which is well known to have 20 kinds of crystalline polymorph (ice Ih, ice Ic, ice II ∼ XIX) (Gasser et al., 2021). Particularly, the investigation of the growth process of interfacial water is extremely challenging, which requires non-invasive high resolution imaging, as the metastable or intermediate edge structures involved are rather fragile. With high sensitivity to the local environment and short range forces, AFM is very useful to detect the structure and dynamics of water molecules on a diversity of substrates, from insulating to metal substrate (Peng et al., 2018a). Until now, AFM imaging, together with *ab initio* calculation, classical molecular dynamics (MD) calculation and AFM simulation, has been applied to the investigation of the interfacial water on a wide variety of substrates, such as minerals (Putnis, 2014), biomolecules (Maver et al., 2016; Dufrene et al., 2017), and organic films (Verma and Sharma, 2010). Previously, we introduced the development of AFM technique both in experiments and simulations, as well as their applications in the study of interfacial water, with particular emphasis on the detection of the nanoclusters and one-dimensional chains (Cao et al., 2019). As a continuation, this perspective reviews the latest progresses in the utilization of AFM imaging in 2D and 3D interfacial water, including adsorbed water networks and electrolytes, as well as their growth processes. An outlook of future AFM study and a brief summary are presented in the end.

## The Ultrahigh-Resolution Imaging of Interfacial Water on Various Substrates

Interfacial water molecules on solid surfaces could form various structures, which depends not only on the lattice spacing and symmetry, but also on the substrate chemical reactivity and water-substrate interaction (Hodgson and Haq, 2009). With the consideration of the lattice matching with bulk water, the close-packed surfaces of Ni, Pt, Ru and Au are ideal substrates for studying water adsorption (Thurmer and Nie, 2013; Shiotari et al., 2019; Ma et al., 2020). Besides, the structures and properties of interfacial water in ionic hydration systems, which are susceptible to the influences of ions in the environment, are of particular importance to tackle many scientific and technical issues (Strmcnik et al., 2009), for instance, the kinetics of hydrogen evolution and oxidation reactions. In the following, we discuss the recent advances in the structural and dynamic properties of interfacial water and electrolytes on various substrates with the application of AFM.

### Characterization of 2D Ice on Various Substrates

1) Characterization of 2D Water Networks on Ni(111)

**FIGURE 1:**
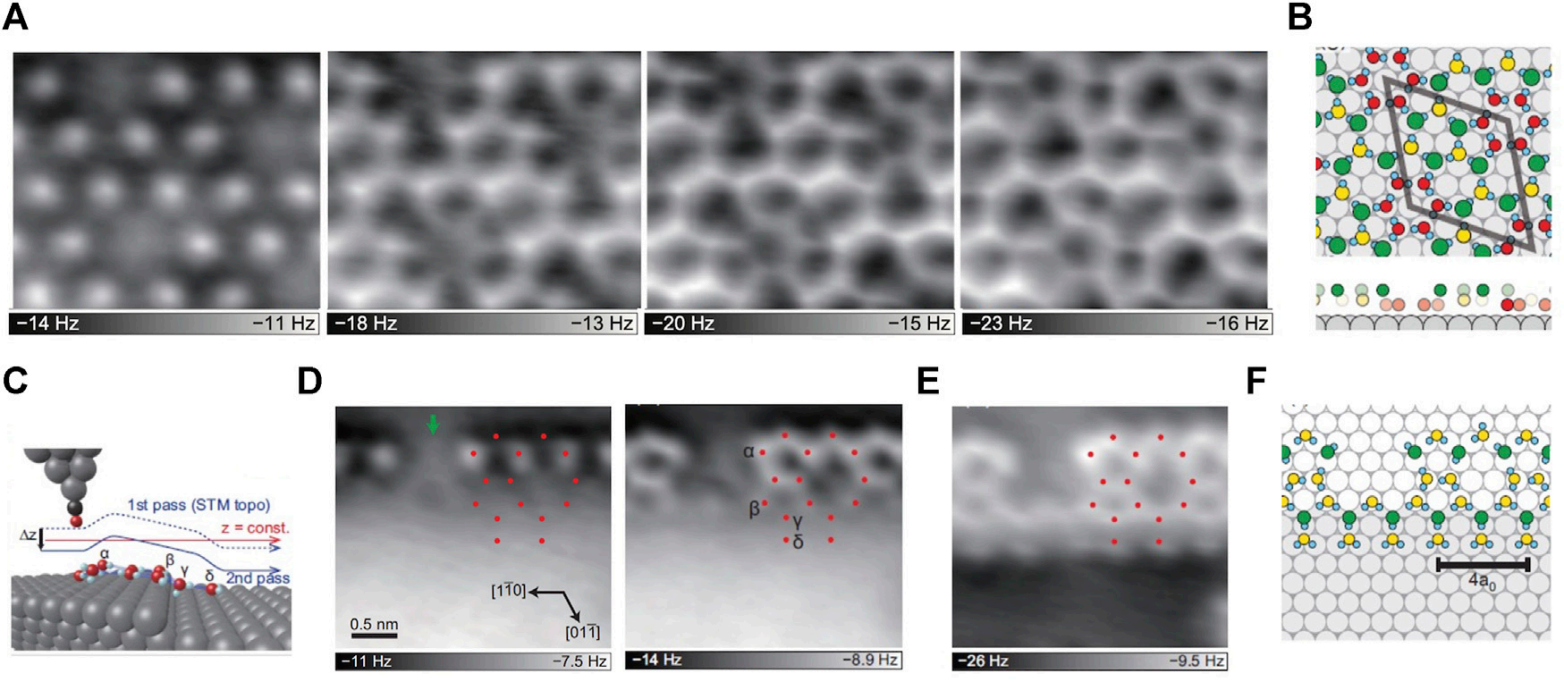
2D water networks on Ni(111) **(A)** AFM images of the 2D water networks of the same area on the terraces, the tip height decreases from left to right **(B)** The proposed structure of the water monolayer based on the AFM images. The gray, cyan, green, yellow and red spheres represent Ni, H, topmost O, middle O and bottommost O atoms, respectively; the unit cell of (√28 × √28) R19 is represented by the gray rhombus; the bottom panels are the side-view of the above structures, without H atoms for clarity **(C)** The schematic diagram of the tip and step. The gray, cyan, black and red spheres represent Ni, H, C and O atoms, respectively **(D)** Constant-height AFM images of the water network at the step edge, the tip height decreases from left to right **(E)** AFM image of the same region in **(D),** but the tip trajectory is consistent with that of the STM image, as the solid blue line in (**C)**. The red dots in **(D–E)** represent the O-atom positions **(F)** The water network structure at the step edge proposed based on the AFM images. Adapted with permission from Shiotari, et al., 2019. *Physical Review Materials* 3(9): 093,001 (Shiotari et al., 2019).

Ni commonly used in alloys and cell electrodes, direct observations of water adsorptions on Ni-based materials are helpful to understand various surface phenomena, such as corrosion, wetting, electrochemical reactions and the water-related heterogeneous catalysis (Michaelides et al., 2004). Recently, with low-temperature AFM, the structures of 2D water networks on the terraces and at the step edges of Ni(111) were observed (Shiotari et al., 2019). It was found that at 78K, water molecules are assembled in disorder, while at 140–145K, they rearrange the structure and form small clusters with a central cyclic (H_2_O)_6_, which are possibly the nucleus of the initial growth of H-bonding networks (Nakamura and Ito, 2004). Such small clusters then disappear after annealed at 150K, homogeneous huge islands of the (√28 × √28)R19° superstructure starting to dominate on the terraces, which are composed of pentagonal, hexagonal and heptagonal rings (Figures 1A,B). Meanwhile, at the monoatomic step edge of Ni(111), another water network is formed, containing pentagonal and octagonal rings along the step direction with a periodicity of four times Ni interatomic distance (4a_0_) (Figures 1C–F). Such a periodic 1D pentagonal-octagonal network closely resembles the domain boundaries of H_2_O/Ru (0001) (Maier et al., 2016), the water islands on stepped Cu(551) surface (Lin et al., 2018), and the defect rows in the second water layer on SnPt (111) (Gerrard et al., 2019). This indicates that the 1D pentagonal-octagonal network structure is a representative defect existing in the interfacial water layers on metal surfaces. Moreover, 2D water networks on metal surfaces, such as Pt (111) (Nie et al., 2010) and Ru (0001) (Maier et al., 2014), have also been studied by AFM. Variations in water networks on different surfaces have been reported, ranging from close-packed metal surfaces to the insulating surfaces. These studies revealed the adsorption structures of water on different metals, which is important for a deep understanding of the catalytic reactivity and the wettability on terraces and at step edges.

2) Determination of Intrinsic Reconstruction of Ice-I Surfaces on Rh (111) and Pt (111)

Compared with non-contact AFM using quartz tuning fork sensors (Thurmer and Nie, 2013), AFM with a silicon cantilever and optical interferometer possesses a higher sensitivity in interatomic force detection (Kaiser et al., 2007). Based on this technique, Kawakami et al. studied the atomic structures of ice-I surfaces on Rh (111) and Pt (111), and found that the AFM images of the terraces of ice on both Rh (111) and Pt (111) have rather similar characteristics, e.g., random distribution, number reduction and (2 × 2) order of dangling H atoms, and (2 × 2) order distortion of O lattice (Kawakami et al., 2020). However, only one crystalline phase of ice (ice-Ih) is observed on Rh (111), while the crystalline phase of ice on Pt (111) varies with the thickness of the ice film, e.g., the number of ice layers (Thurmer and Nie, 2013). That is to say, it is ice-Ic at medium thickness (10–50 BLs) and ice-Ih in a dominant state at other thicknesses. Although the crystalline phase of ice on Pt substrate differs at different ice thickness, their densities of the dangling H atoms are the same, and their AFM images are quite similar with a (2 × 2) order. This reveals that the changes in the H-atom orientation and the interface effects of ice Ih and ice Ic have a negligible influence on the surface reconstruction. Moreover, it is found that the (2 × 2) order reconstruction of ice-I surfaces, including the sparse distribution of the residual dangling H atoms and the removal of dangling H atoms, is caused by the electrostatic repulsion between the dangling H atoms. The above findings about the molecular reconstruction are crucial to understand chemical and physical phenomena, such as the growth and melting of ice, and chemical reactions (Kawakami et al., 2020).

3) Atomic Imaging of Edge Structure and Growth of a 2D Hexagonal Ice

**FIGURE 2:**
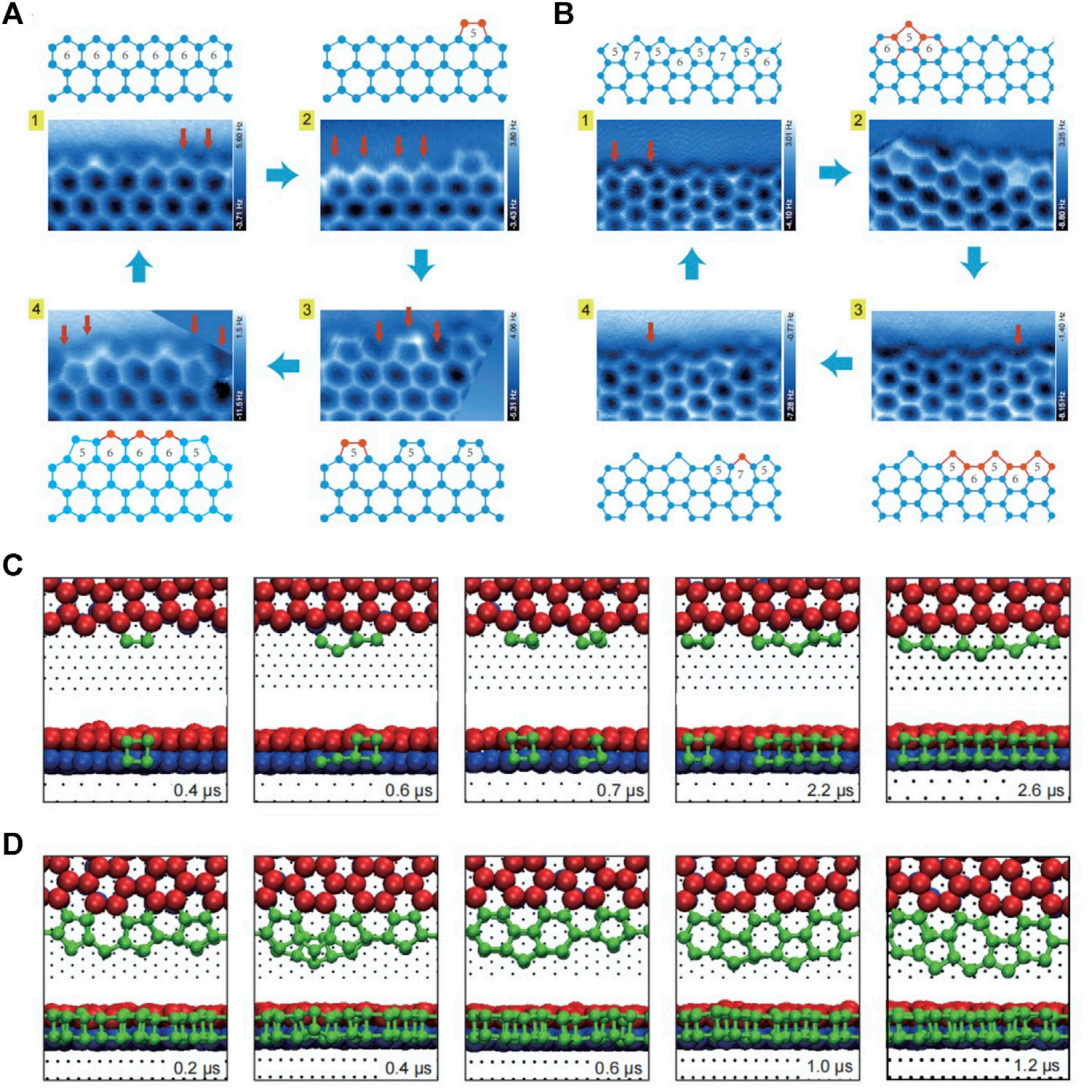
The growth process of zigzag and armchair edges of the 2D bilayer hexagonal ice formed on Au(111) **(A–B)** Constant-height AFM images and corresponding models of the steady state (1) and metastable states (2–4) of the zigzag **(A)** and armchair edges **(B) (C–D)** Time-elapsed snapshots during the growth of zigzag **(C)** and armchair edges **(D)** obtained by MD simulation. In **(A–B)**, the proposed growing process (from one to 4) is indicated by blue arrows; the addition of one bilayer water pair is labeled by a red arrow in AFM images; the red balls and sticks in ball-stick models represent the newly added bilayer water pairs, while the blue ones represent the existing structures. In **(C–D)**, the simulation times are shown on bottom right of each snapshot, the bottom- and top-layer water molecules of the preexistent bilayer ice are denoted by the blue and red spheres, respectively; the newly deposited water molecules are denoted by the green spheres. Adapted with permission from Ma, et al*.* 2020. *Nature* 577(7788): 60–63 (Ma et al., 2020).

Although the structure of 2D water network on surface has been widely studied (Nie et al., 2010; Kiselev et al., 2017), it is rather challenging to capture the atomic details of metastable or intermediate edge structures during ice growth. This is due to the fact that the fragileness and the short life span of intermediate edge structures call for fast and non-invasive detection (Lupi et al., 2014). Recently, Ma et al. achieved a high resolution imaging of the edge structures of a 2D bilayer hexagonal ice on Au (111) by weakly perturbative AFM with a CO-functionalized tip (Ma et al., 2020). Combining experiments with theoretical calculations, the orientation of each water molecule, namely the O-H directionality in the 2D bilayer ice, was revealed. Moreover, a new type of edge, which coexists with the zigzag edge in 2D hexagonal ice and along the armchair direction but is reconstructed into a complex periodical structure consisting of 5756-membered rings, was also found. Ma et al. also achieved the freeze and imaging of various intermediate structures at the two types of edges, and rebuilt distinct ice growth mechanisms (Figures 2A,B). Combined with *ab initio* calculations and classical MD simulations, the growth at the zigzag edge was found to follow a collective bridging mechanism facilitated by a periodic array of pentagons, in contrast to a local seeding growth mode with a manifestation of the 5756–5656 interconversion at the armchair edge (Figures 2C,D). Furthermore, not only the distance between water molecules but also the commensurability between ice lattice and the substrate was found to have a negligible effect on the relative stability of the two types of edges, demonstrating the generality of observed growth behavior. Such new growth pattern is in sharp contrast to the conventional growth mode of bilayer hexagonal ices and 2D hexagonal material, shedding light on the physical mechanism of ice growth on hydrophobic substrates or under hydrophobic confinement.

### Probing the Adsorption of Electrolytes at Liquid/Kaolinite Interfaces

The above mentioned works were all performed in a vacuum at low temperature using *non-contact* AFM (nc-AFM) to guarantee that the sample would not be disturbed and the tip would not be contaminated. However, in case of structure probing of the solid/liquid interface at room temperature, nonnegligible disturbance and tip contamination becomes unavoidable. As a result, nc-AFM imaging under such conditions becomes very difficult. Therefore, the contact mode AFM, whose tip contacts the measured sample during imaging, is more appropriate here due to its higher stability in such cases. With the contact mode AFM, surface structures in liquids could be characterized.

Adsorption of electrolytes (ions) at liquid/solid interfaces plays a critical role in nanomaterial processing to gain desired structures and morphology, since it can alter physicochemical nature of materials (Nelson and Schwartz, 2013; DeWalt-Kerian et al., 2017). Various electrical double layer (EDL) models have been used to describe the ion adsorption on charged surfaces in liquids, but the model-dependent approaches lead to inaccurate predictions of ion adsorption due to inevitable assumptions, for example, whether the diffusion and thermal motion of ions are taken into account for molecular modeling (Modi and Fuerstenau, 1957; Ardizzone et al., 1982). Without any approximation or assumption, Chang et al. studied the electrolytes adsorption at liquid/kaolinite interfaces using an *in situ* AFM (Chang et al., 2021). They directly visualized the ions located in the EDL and adsorbed on the anisotropic kaolinite, and detected the changes of surface structure of kaolinite during the adsorption of mono- and divalent metal cations.

As a result, it was found that the adsorption of K^+^ and Cl^−^ in solution cannot change the lattice structure of kaolinite surfaces. Unlike monovalent ions, the adsorption of calcium ions can lead to the changes of the surface morphology at high pH, presenting in a specific adsorption mode. This suggests the strong and direct binding between cations and substrates. Moreover, it was first found that the specific adsorption caused by the hydrolysis of Ca^2+^ can weaken the electrostatic interaction and decrease the ionic charge. To conclude, AFM is a very useful imaging tool to disclose ion adsorption at the liquid/kaolinite interface and it can be extended to other interfaces.

## Perspective

### Identification of Multiple Chemical Species in 2D H-Bonding Networks

Identification of multiple chemical species such as OH^−^ or H_3_O^+^ solvated in 2D and 3D H-bonding water networks is significant for deepening our knowledge about electrochemical reactions, such as hydrogen or oxygen evolution reaction. Previous studies of the systems like partially dissociated water networks or hydrated protons solvated in H-bonding networks, have been mainly investigated by vibrational spectroscopy such as X-ray photoelectron spectroscopy, infrared and Raman spectroscopies, which can determine the extent of partial dissociation (Meier et al., 2018), or the molecular structures of hydrated protons in water clusters (Fournier et al., 2015). However, these techniques suffer from the difficulty of spectral assignment and the limited spatial resolution (Shen and Ostroverkhov, 2006). In contrast, nc-AFM with a CO-terminated tip which can be operated in a vacuum at low temperatures, shows the ability to imaging the water molecule with sub-molecular resolution in a nearly non-invasive manner (Peng et al., 2018a; Peng et al., 2018b). In this way, partially dissociated water networks on various substrates, like Fe_3_O_4_(001) (Meier et al., 2018) and Cu(110) (Shiotari and Sugimoto, 2017) have been studied. However, the identification of chemical species related to water just based on the AFM images is difficult due to the fact that AFM is not very sensitive to H atom, which is small and interacts weakly with the tip. Fortunately, this difficulty can be overcome by the aid of DFT calculation and AFM image simulations (Hapala et al., 2014). Nevertheless, due to the high similarity of H_3_O^+^ to H_2_O, the imaging and identification of H_3_O^+^ in the H-bonding network of water is much more challenging, and the related AFM-based studies have not been reported yet. It is believed that by further improving the resolution and sensitivity of qPlus-AFM, this will soon no longer be an issue.

### 3D Atomic Force Microscopy

Interfacial aqueous layer plays an intermediate or significant role in many aspects, such as corrosion, nanopatterning and adhesion in material science (Garcia et al., 2014), protein folding, inactivation and molecular identification in molecular biology (Laage et al., 2017), the dissolution and growth of ore in geology (Laanait et al., 2015). To fully understand such phenomena and reveal the detailed interactions of liquid water on different solid interface, the detailed 3D structure of hydration layer needs to be established. While nc-AFM is usually affected by the motion of liquid molecules, 3D-AFM with an authentic 3D depth and a high spatial resolution of 1–3 Å is the newest and most successful attempt to achieve this goal, making an accurate imaging of the atom position. In fact, it has already been applied to uncover the nature of a few liquid/solid interfaces, achieving high resolution imaging of water layers at the flat liquid/mica interface (Fukuma et al., 2010) and on non-flat surfaces, such as lipid membranes (Kobayashi et al., 2013), proteins (Herruzo et al., 2013), or DNA (Kuchuk and Sivan, 2018).

The achievements of 3D-AFM made in the investigation of interfacial water on stiff crystalline surfaces and in soft biomolecules (Fukuma and Garcia, 2018) highlight its widely potential applications in catalysis, electrochemistry, cell and molecular biology. Nevertheless, there are still some scientific and instrumental challenges for the promotion of 3D-AFM. For instance, the 3D-AFM-based z-depth imaging, with a maximum of 10 nm (Martin-Jimenez et al., 2016) and a common value of 1 nm, is ideal for the exploration of the adsorption of ions and solvent molecules on flat substrates (Jariwala et al., 2017), but it is rather small for detecting the hydration structures on non-flat systems, such as isolated proteins, nanostructured surfaces and cells (Fukuma and Garcia, 2018). Thus, it is of great significance to improve the sensitivity, resolution and scanning speed of 3D-AFM for its broader applications, e.g., the study of 3D distribution of flexible surface structures, such as chain polymers or lipid headgroups on a biological membrane surface, and *in-situ* observation.

### Multifunctional AFM Techniques for Aqueous Battery Characterization

Being capable of performing under realistic *in-situ* conditions, multifunctional AFM is conductive to revealing the detailed mechanisms of scientific phenomena and technological processes, the extremely important evolution of liquid/solid interface in battery, for instance. At present, different kinds of multifunctional AFMs have been used in the research of lithium-ion batteries with organic solvents as electrolytes (Yoon et al., 2016), among which electrochemical AFM (EC-AFM) was more widely utilized than others. It can characterize the morphology evolution on anodes (Jeong et al., 2001) and cathodes (Wu et al., 2017), the formation of Li-dendrite (Shen et al., 2018) and the kinetic process of solid electrolyte interface (Novak et al., 2001). Electro-chemical strain microscopy is a very useful tool to measure the volumetric strain driven by potential pulse, and to detect the microscopic details of electronic or ionic transport processes (Yang et al., 2015). Meanwhile, the ability to detect the surface potential between the tip and the sample measured, allows Kelvin probe force microscopy to investigate the relationship between ion distributions and battery performance, as well as the impact of surface charges on ionic intercalation (Luchkin et al., 2014). Moreover, combined with infrared or Raman spectroscopy and mass spectrometry, AFM can effectively overcome the shortcoming of being unable to distinguish the composition of the detected substances, greatly expanding its application in energy fields.

Aqueous battery with water as the electrolyte solvent is the most potential technology for large-scale energy storage systems of wind and solar photovoltaic power generation (Wang et al., 2018). This is due to its superiority of low cost, environmental friendliness, high safety and theoretical capacity (Glatz et al., 2019), while the application of lithium-ion batteries is hindered by its high cost, low safety and environmental issues. However, studies of aqueous batteries are still at the early stage, and further investigations need to be carried out, including in-depth structures and dynamic properties of aqueous battery, as well as their changes in the electro-chemical processes (Zhou et al., 2020). Despite their powerfulness in nanoscale characterization, all types of multifunctional AFMs have limitations in spatial and temporal resolution. For example, EC-AFM has limited scanning rate, thus some fast interface reactions cannot be monitored in real time. In addition, drift during scanning make it difficult to obtain reliable surface characteristics (Chen et al., 2020). These limitations are expected to be overcome in the near future by developing low-noise cantilever deflection sensor (Fukuma et al., 2005) and drift-compensated data acquisition schemes (Abe et al., 2007). Besides, optimal tip preparation is often a key issue in AFM-based techniques such as tip enhanced Raman spectroscopy to ensure high enhancement and good reproducibility (Toca-Herrera, 2019).

### Machine Learning to Enhance AFM Interpretation

The interpretation of AFM signals, especially the 3D features appearing in the submolecular-resolution 3D-AFM images, has been achieved by introducing MD simulations. In 2015, with the comparison between the result of 3D-AFM experiment and MD simulation, it was found that the activities between the uppermost surface atoms and tip apex predominantly affected the tip-sample interaction (Fukuma et al., 2015). This finding provides a qualitative answer to the question on the imaging mechanism: why the intrinsic 3D hydration structure (without the AFM tip) is similar to the sub-nanometer resolution contrast appearing in 3D-AFM images.

However, MD simulation has been mainly applied to study the pure water on uniform and rigid surfaces (Miyazawa et al., 2016; Vilhena et al., 2016). It is impractical to utilize MD to analyze 3D-AFM features in many complex problems, owing to its high computational costs. Currently, there is still a large gap between the AFM simulation and experiments. Many issues under more complicated conditions remain to be tackled theoretically, such as the treatment of ions, the influence of ions on force contrast, and the appropriate construction of tip model. In recent years, with the emergence of more and more powerful, effective and efficient algorithms for object detection, classification, image segmentation and quality enhancement, machine learning (ML) has been gradually employed in many physical (Karniadakis et al., 2021) and chemical (Mater and Coote, 2019) investigations, including the applications in solving related questions about AFM.

Using convolutional neural networks, Gordon et al. discerned several spatially correlated patterns of self-organized nanoparticles based on the mixed, highly varied experimental AFM images (Gordon et al., 2020). What’s more, Alldritt et al. developed a deep learning framework that enables a unique molecular structure descriptor to match a suit of AFM images, allowing direct structure determination of organic molecules based on AFM images (Alldritt et al., 2020). Going even further, applying ML to identify configurations and structural evolution of larger and more complex systems, like liquid and ionic hydration layer, requires a great deal of effective, high quality training data obtained from simulation or experiment. As a result, it will lead to the need for a delicate balance between accuracy increasing and resource consuming. Nevertheless, ML is still a promising tool for the in-depth study of all kinds of complex water-related systems, where traditional interpretation means have been a failure or cannot even be attempted. In other words, it could save researchers from tedious and time-consuming repetitive work, and uncover hidden patterns or properties that are invisible to the human eye. Therefore, the development of ML technique will play a crucial role in prompting AFM at the forefront of the characterization technologies.

## Conclusion

This perspective presents recent applications of AFM in the study of 2D and 3D interfacial water on solid interfaces, including structures of water networks, ice reconstructions, edge structures and growth mechanism of 2D hexagonal ice as well as the adsorption of electrolytes. The prospect of AFM is to study the more intricate and subsistent interfacial water-related systems, which needs to employ 3D- and multifunction AFM. Furthermore, the interpretation of AFM images of the intricate liquid/solid interface, especially the evolution of 3D-AFM features in AFM image, is extremely challenging due to the fact that AFM signals can be affected by environmental factors. Thus, it urgently requires a further development of effective interpretation approaches of AFM. ML is the most likely solution for the related questions about AFM imaging in complex systems associated with water, when traditional interpretation methods fail or cannot even be attempted. It is reasonable to predict that, with the improvement of AFM imaging and ML techniques, there will be a rapid increase in the atomic-scale investigations of the structural and dynamic properties of liquid/solid interfaces in the foreseeable future.

## Data Availability

The original contributions presented in the study are included in the article/Supplementary Material, further inquiries can be directed to the corresponding authors.
